# Association between Serum Ferritin Levels and Risk of the Metabolic Syndrome in Chinese Adults: A Population Study

**DOI:** 10.1371/journal.pone.0074168

**Published:** 2013-09-16

**Authors:** Jiang Li, Rui Wang, Dan Luo, Shuang Li, Cheng Xiao

**Affiliations:** 1 Department of Laboratory Medicine, China-Japan Friendship Hospital, Ministry of Health, Beijing, China; 2 Institute of Clinical Medicine, China-Japan Friendship Hospital, Ministry of Health, Beijing, China; 3 Blood Screening Laboratory, Beijing Red Cross Blood Center, Beijing, China; 4 Department of Rheumatology, The People’s Hospital of Yichun city, Yichun, Jiangxi Province, China; Penn State Hershey Medical Center, United States of America

## Abstract

Ferritin is a ubiquitous intracellular protein that can store and release iron and act as a buffer against iron deficiency and iron overload. Ferritin is widely used as a clinical biomarker to evaluate iron status. Increased serum ferritin concentrations have been reported to be associated with metabolic syndrome (MetS) features. However, serum ferritin concentrations differ significantly according to sex and ethnicity, and the data concerning the relationship between serum ferritin concentrations and MetS in Asian men and women are conflicting. This study aimed to explore the relationship between serum ferritin and MetS in Chinese population. Fasting blood samples and anthropometric data collected on 8,441 adults aged 18 and older in 2009 as part of the China Health and Nutrition Survey, a large-scale longitudinal, household-based survey in China. Data was collected by trained physicians and biomarkers were measured with Hitachi Clinical Autoanalyzer 7600 D model and P model. Median levels of serum ferritin were significantly higher in men compared with women (121.9 vs. 51.0 ng/ml, P < 0.001), and significantly lower in non metabolic syndrome population with MetS population (73.2 vs. 106.0 ng/ml, P < 0.001). The difference remained significant after further adjusted for age, nationality, Body mass index (BMI), smoking status, and alcohol consumption. For both men and women, the highest prevalence of MetS occurred in the highest quartile of serum ferritin. The odds ratios increased progressively across the ferritin quartiles (P<0.001 for trend). Increased serum ferritin concentrations are associated with the metabolic syndrome among men and women in China.

## Introduction

Iron, a necessary trace element that participates in many biological oxidations and accumulate in tissue, can lead to pathology change in the liver, heart, endocrine organs, and musculoskeletal system [1,2]. Some studies in patients with hemochromatosis or hematologic diseases indicated that increased accumulation of iron affects the synthesis and secretion of insulin by the pancreas [3,4]and compromises insulin action in target tissues [5-7]. Therefore, it contributes to the development of insulin resistance. Ferritin is a ubiquitous intracellular protein that can store and release iron and act as a buffer against iron deficiency and iron overload. Ferritin is widely used as a clinical biomarker to evaluate iron status and especially important for detecting iron deficiency.

MetS is a combination of medical disorders that closely link to insulin resistance and increases the risk of cardiovascular disease [8-10]. Increasing evidence has shown that body iron excess is associated with one or more MetS components [11-15]. Even moderately increased iron stores, represented by high-normal ferritin concentrations, are associated with adverse health conditions, such as hypertension [16], dyslipidemia [17,18], elevated fasting insulin and blood glucose [19-22], and central adiposity [23]. Chul-Hee Kim and colleagues reported that increased serum concentrations of ferritin were only associated with metabolic syndrome among men but not among women in Korean population. Nicola Martinelli et al [24] ,also found that ferritin was the only significant independent predictor of hepcidin in males. To date, only a few studies on ferritin and MetS conducted in special Chinese population [11,25,26] or other Asian population [21]. As serum ferritin concentrations differ significantly among age, sex, and race [27,28], we explore the association between serum ferritin concentrations and MetS among a free-living Chinese population by using a representative, randomly selected sample from nine provinces across China.

## Methods

### Ethics statement

All the documentations & procedures comply with GCP & Human Ethics Protocol Rules and Related Chinese Laws. CHNS project was approved by the Human & Clinical Research Ethics Committee of China-Japan Friendship Hospital. Participants who signed blood consent form in the study were required to fast overnight (at least 8 hours) before the blood collection by trained phlebotomists under standard protocol.

### Study Population

The data collected from the China Health and Nutrition Survey (CHNS), a large-scale longitudinal, household-based survey in China. The CHNS has followed individuals randomly selected from 228 communities since 1989 and designed to represent a set of large provinces with a range of economic and demographic variation, covering approximately 56% of China’s population, including Heilongjiang, Liaoning, Shandong, Henan, Hubei, Hunan, Jiangsu, Guangxi, Guizhou (from north to south) [29-31]. A multistage, random cluster process was used to draw the surveyed sample. Counties in the nine provinces were initially stratified by income (low, middle, and high) and a weighted sampling scheme was used to randomly select four counties in each province. A higher income city and a lower income city within each province were selected. In addition, the township capital and three villages within the counties were randomly selected. Finally, within each city, urban and suburban neighborhoods were randomly selected. In each community, 20 households were interviewed. The 2009 CHNS sample consists of 216 communities from 9 provinces, comprising of 36 urban neighborhoods, 36 suburban neighborhoods, 36 towns and 108 villages. Details about the study design and sampling strategies are available at the World Wide Web site (http://www.cpc.unc.edu/projects/china/home.html) and somewhere else [32,33].

In total, 8,641 fasting blood samples were drawn from participants aged 18 and older. 200 participants who did not have serum ferritin result due to a serious haemolytic state or did not have MetS components information were excluded in the analysis. Therefore, we analyzed the association between serum ferritin concentrations and MetS among 8,441 participants aged 18 and older.

### Data collection methods

Trained interviewers (physicians and nutritionists) collected questionnaire-based demographic, anthropometric, and lifestyle data from each participant, including date of birth, gender, ethnicity, occupation, education level, health status, health behavior (smoking habit, alcohol use, physical activity), food and beverage intake, tea intake, diseases under current or previous treatment, use of drugs and supplements parental history of selected diseases, blood collection control information (collection time, treatment time, transfer time and storage time). In analysis, smoking status was grouped as non-smokers and smokers based on reported cigarettes consumption. Alcohol drinking was classified based on reported consumption frequencies. Education level was categorized into three groups (primary school or below, middle and high school, and above high school).

Height and weight were measured directly by trained interviewers who followed standard protocols similar to the National Health and Nutrition Examination Survey (NHANES) protocol developed by the National Center for Health Statistics in the the United States of America. Weight in lightweight clothing was measured to the nearest 0.1 kg and height was measured to the nearest 0.1 cm. BMI was calculated as weight in kg divided by height in square meters. All interviewers took part in interobserver reliability testing as part of training. Complete anthropometric data were obtained during a physician-conducted physical examination [31].

Participants who signed blood consent form in the study were required to fast overnight (at least 8 hours) before the blood collection by trained phlebotomists under standard protocol. Blood was drawn from an antecubital vein in the morning and was transferred to local hospital for further treatment within 2 hours of blood collection. Blood specimens were collected in a 4-ml EDTA vacuum tube for routine examination and two 4-ml separation gel vacuum tubes for biochemical analysis, which were stored in icebox in the field. When specimens were transferred to local hospital, they were centrifuged at 3000g for 10 minutes at room temperature as soon as possible and separated into 9 aliquots. Except the samples for field test, other samples were storage in -80 degree freezers.

The fasting serum glucose (enzymatic method) and blood routine examination were measured at local hospitals. The calibrator and control serums were provided by the Department of Laboratory Medicine of China-Japan Friendship Hospital (CJFH) in same lot number. Other biochemical markers such as fasting serum high-density lipoprotein cholesterol (HDL-C), low-density lipoprotein cholesterol (LDL-C), triglyceride (TG), total cholesterol (TC) and high-sensitivity C-reactive protein (hs-CRP) were analyzed by an automatic clinical chemistry analyzer (Hitachi 7600 D and P model, Japan) at the Department of Laboratory Medicine of CJFH. The serum concentration of HDL-C, LDL-C ,TG and TC was determined by an enzymatic colorimetric method (Kyowa,Japan). The concentration of fasting serum insulin and ferritin was determined by a commercial Radioimmunoassay Kit (Beijing North institute of biological technology, China). Insulin resistance was estimated with a Homeostasis Model Assessment (HOMA-IR) equation. The concentration of high-sensitivity C-reactive protein (hs-CRP) was measured by immunoturbidimetric immunoassay method (Denka Seiken, Japan).

MetS was defined based upon the updated the Adult Treatment Panel III of the National Cholesterol Education Program (NCEP-ATPIII) for Asian-Americans [34], which recognizes the existence of MetS if any 3 or more of the following 5 components are present: 1. Abdominal obesity: waist circumference ≥ 90 cm in men or ≥ 80 cm in women; 2. Hypertriglyceridemia: TG ≥1.7 mmol/L(≥ 150 mg/dl); 3. Low HDL cholesterol: HDL-C< 1.03 mmol/L in men or 1.3 mmol/L in women(

< 40 mg/dL for men, < 50 mg/dL for women); 4. High blood pressure: Blood pressure ≥ 130 mm Hg SBP or 85 mm Hg DBP; 5. High fasting glucose: Fasting glucose ≥ 6.1 mmol/L(≥ 110mg/dL).

World Health Organization (WHO)’s hemoglobin thresholds used to define anemia. Hemoglobin concentration less than 130 g/L for men and less than 120 g/L for women [35]. Iron deficiency anemia was defined as the presence of both anemia and a plasma ferritin concentration less than 15 ng/ml [36].

### Statistical analysis

The current analysis was restricted to 8,441 subjects aged ≥18 years who had complete ferritin and MetS data. For baseline characteristics of participants, means (±SD) or median (interquartile range) were used for continuous variables, and counts and percentages for categorical variables. Unpaired t-tests were used to compare continuous variables, and chi-square tests to compare categorical variables between sex groups. Because the data were skewed, we evaluated the significance of any differences in median values between groups using Mann-Whitney U test (nonparametric test).

To examine whether differences of ferritin levels in MetS population and non-MetS population were independent of age, BMI, systolic blood pressure (SBP), smoking use, alcohol consumption, and nationality, we performed linear-regression analyses with Log-transformed ferrtiin as the dependent variable. Multivariable logistic regression analyses were performed to examine the association between differences of ferritin levels and the prevalence of MetS and its components, The odds ratios (ORs) (95% CI) for the prevalence of MetS and its components were calculated for quartiles of serum ferritin, with the lowest ferritin quartile as the reference. Tests for trend across quartiles were computed by including a variable with the median value for each quartile as a continuous variable in the logistic regression models. All the reported P values were 2-tailed, and those <0.05 were considered to be statistically significant. The odds ratios of all components were adjusted for age, nationality, smoking use, alcohol consumption and hs-CRP level in different regression models. Statistics analysis was performed with SAS 9.2 (SAS Institute, Cary, North Carolina).

## Results

The baseline characteristics and the descriptive statistics of the study population are summarized in Table 1. The median age was 51.1 years (range, 18-99 years). Compared with women, men had higher waist circumference, SBP, diastolic blood pressure (DBP), TG, fasting plasma glucose (FPG), and lower TC, HDL-C and LDL-C. Man had a significantly higher rate of current smoking (54.9% versus 3.6%) and alcohol consumption (59.8% versus 8.8%) than women. The prevalence of the MetS was 19.9% in men and 25.4% in women.

**Table 1:** Baseline characteristics of participants studied.

	**Total (n=8441**)	**Men (n=3939**)	**Women (n=4502**)	**Z for gender comparison**
Age,years	51.1 (24.5-75.9)	51.2 (24.2-76.0)	51.0 (24.8-75.9)	0.65
BMI^a^, kg/m2	23.1 (18.2-29.6)	23.1 (18.3-29.4)	23.0 (18.2-29.7)	0.80
Waist circumference, cm^b^	82.0 (66.5-100.1)	84.0 (68.0-102.0)	80.3 (65.2-99.0)	<0.001
SBP, mm Hg^c^	120.7 (100.0-160.0)	122.0 (100.7-160.0)	120.00 (97.3-161.3)	<0.001
DBP, mm Hg^c^	80.0 (62.0-100.0)	80.7 (66.0-100.7)	80.0 (60.7-100.0)	<0.001
TC, mmol/L	4.8 (3.4-6.6)	4.7 (3.4-6.5)	4.8 (3.4-6.7)	<0.001
HDL-C, mmol/L	1.4 (0.9-2.1)	1.3 (0.9-2.1)	1.4 (1.0-2.1)	<0.001
LDL-C, mmol/L	2.9 (1.6-4.6)	2.9 (1.5-4.5)	2.9 (1.7-4.7)	<0.001[37]
TG, mmol/L	1.2 (0.6-3.6)	1.3 (0.6-4.1)	1.2 (0.6-3.3)	<0.001
FPG, mmol/	5.1 (4.2-7.5)	5.1 (4.1-7.8)	5.1 (4.2-7.2)	<0.001
hs-CRP, n(%)				<0.001
Level-1(< 3 mg/l)	6403 (75.86)	2991 (75.93)	3412 (75.79)	
Level-2(3-10 mg/l)	1692 (20.05)	764 (19.40)	928 (20.61)	
Level-3(> 10 mg/l)	346 (4.10)	162 (3.60)	184 (4.67)	
Smokers, n (%)				<0.001
Never	5840 (69.2)	1517 (38.5)	4323 (96.0)	
Former	274 (3.3)	259 (6.6)	15 (0.3)	
Current	2327 (27.5)	2163 (54.9)	164 (3.7)	
Alcohol use/last year, n (%)				<0.001
No	5691 (67.4)	1583 (40.2)	4108 (91.3)	
Yes	2750 (32.6)	2356 (59.8)	394 (8.7)	
Education, n (%)				<0.001
Low	3655 (43.3)	1337 (33.9)	2318 (51.5)	
Medium	2787 (33.0)	1495 (38.0)	1292 (28.7)	
High	1999 (23.7)	1107 (28.1)	892 (19.8)	
History of diabetes	907 (10.8)	458 (11.6)	449 (10.0)	0.01
History of cardiovascular disease	82 (1.0)	39 (1.0)	43 (1.0)	0.87
History of stroke	117 (1.4)	79 (2.0)	38 (0.8)	<0.001
History of hypertension	1114 (13.2)	500 (12.7)	614 (13.6)	0.20
ATP III MetS, n (%)	1926 (22.8)	783 (19.9)	1143 (25.4)	<0.001
Waist circumference (>90 cm in men and >80 cm in women)	3633 (43.0)	1189 (30.2)	2444 (54.3)	<0.001
Triglycerides (1.7mmol/L)	2462 (29.2)	1243 (31.6)	1219 (27.1)	<0.001
Glucose (>5.6mmol/L)	1168 (13.8)	618 (15.7)	550 (12.2)	<0.001
HDL (<1.03mmol/L in men and <1.3mmol/L in women)	2167 (25.7)	663 (16.8)	1504 (33.4)	<0.001
Hypertension(>130/85mmHg or treatment)	3517 (41.7)	1816 (46.1)	1701 (37.8)	<0.001

Data are presented as median (5 percentile, 95 percentile) or percent.

Chi-square test for categorical variables, the unpaired t test or Mann-Whitney U test for continuous variables.

BMI, body mass index; SBP, systolic blood pressure; DBP, diastolic blood pressure; TC, total cholesterol; HDL-C, high density lipoprotein cholesterol; LDL-C, low density lipoprotein cholesterol; TG, triglycerides; FPG, Fasting plasma glucose; hs-CRP, high-sensitivity C-reactive protein; MetS, metabolic syndrome.

a Information on BMI was available for 3908 men and 4482 women.

b Information on waist circumference was available for 3898 men and 4461 women.

c Information on SBP and DBP was available for 3937 men and 4499 women.

Median levels of serum ferritin were significantly higher in men compared with women (121.9 vs. 51.0 ng/ml, P < 0.001), and significantly lower in non-MetS population than in MetS population (73.2 vs. 106.0 ng/ml, P < 0.001). The difference remained significant after further adjusted for age, nationality, BMI, smoking status, and alcohol consumption.

Age adjusted partial correlation analysis showed that ferritin is associated with MetS, especially with hyperglycemia and obesity. For both sex groups, the highest prevalence of MetS occurred in the highest quartile of serum ferritin. The prevalence of MetS components, included elevated waist circumference, elevated blood pressure, elevated fasting glucose, elevated triglycerides, and elevated HDL-C, are all increased significantly with increasing serum ferritin in men and women (Table 2). After adjusted for age, nationality, BMI, smoking status, and alcohol consumption, serum ferritin levels remained positively associated with prevalence of MetS in both sex groups.

**Table 2:** Prevalence of the metabolic syndrome and its components by sex- specific quartiles of serum ferritin.

	**Q1**	**Q2**	**Q3**	**Q4**	**P(trend**)
**Men**					
Ferrintin (mg/L)	51.9	97.2	154.0	422.5	
MetS(%)	11.7	15.9	18.8	33.1	<0.001
Waist (%)	22.8	24.9	32.3	42.1	<0.001
TG (%)	18.68	25.4	33.6	48.7	<0.001
HDL (%)	11.4	14.3	16.7	25.0	<0.001
BP (%)	44.2	44.7	46.3	49.3	0.01
GLU (%)	10.7	13.8	15.4	22.9	<0.001
**Women**					
Ferrintin(mg/L)	12.9	36.0	68.4	142.7	
MetS	13.0	19.0	27.3	42.3	<0.001
Waist (%)	43.8	48.6	58.7	68.1	<0.001
TG (%)	15.6	22.0	28.3	42.3	<0.001
HDL (%)	29.1	32.1	32.4	40.0	<0.001
BP (%)	22.6	30.8	45.2	52.8	<0.001
GLU (%)	4.8	9.3	12.1	22.7	<0.001

The odds ratios for MetS increased progressively across the ferritin quartiles (P<0.001 for trend) in women and in men (Table 3). After adjusted for age, nationality, smoking status, alcohol consumption and hs-CRP in different models, except the result of BP in women group, the results remained unchanged.

**Table 3:** Odds ratio (95% CI) and adjusted odds ratio (95% CI) of the metabolic syndrome and its components by sex- specific quartiles of serum ferritin.

		**Q1**	**Q2**	**Q3**	**Q4**	**P (trend**)
**Men**						
Ferrintin(mg/L)		51.9	97.2	154.0	422.5	
MetS		1	1.43 (1.11-1.86)	1.75 (1.36-2.25)	3.75 (2.96-4.74)	<0.001
	model 1^a^		1.49 (1.15-1.93)	1.86 (1.44-2.39)	4.08 (3.21-5.19)	<0.001
	model 2^b^		1.49 (1.15-1.93)	1.86 (1.44-2.39)	4.05 (3.19-5.14)	<0.001
Waist		1	1.12 (0.91-1.38)	1.62 (1.32-1.98)	2.46 (2.02-3.00)	<0.001
	model 1		1.16 (0.94-1.43)	1.70 (1.39-2.08)	2.63 (2.16-3.21)	<0.001
	model 2		1.15 (0.94-1.42)	1.69 (1.38-2.07)	2.60 (2.13-3.18)	<0.001
TG		1	1.49 (1.20-1.85)	2.22 (1.80-2.73)	4.16 (3.39-5.10)	<0.001
	model 1		1.48 (1.19-1.84)	2.18 (1.77-2.69)	4.09 (3.33-5.01)	<0.001
	model 2		1.47 (1.19-1.83)	2.17 (1.76-2.67)	4.06 (3.31-4.99)	<0.001
HDL		1	1.30 (1.00-1.70)	1.56 (1.20-2.02)	2.60 (2.04-3.32)	<0.001
	model 1		1.30 (1.00-1.70)	1.60 (1.23-2.07)	2.65 (2.07-3.39)	<0.001
	model 2		1.30 (1.00-1.70)	1.60 (1.23-2.07)	2.64 (2.06-3.37)	<0.001
BP		1	1.02 (0.85-1.22)	1.09 (0.91-1.30)	1.23 (1.03-1.47)	<0.001
	model 1		1.11 (0.92-1.34)	1.23 (1.02-1.48)	1.46 (1.21-1.76)	<0.001
	model 2		1.11 (0.92-1.34)	1.22 (1.01-1.47)	1.44 (1.19-1.74)	0.0013
GLU		1	1.34 (1.02-1.76)	1.53 (1.17-2.00)	2.48 (1.93-3.19)	<0.001
	model 1		1.42 (1.08-1.87)	1.66 (1.26-2.17)	2.82 (2.17-3.65)	<0.001
	model 2		1.43 (1.08-1.88)	1.67 (1.28-2.19)	2.82 (2.18-3.64)	<0.001
**Women**						
Ferrintin(mg/L)		12.9	36.0	68.4	142.7	
MetS		1	1.58 (1.25-1.98)	2.52 (2.02-3.13)	4.92 (3.99-6.08)	<0.001
	model 1		1.25 (0.99-1.58)	1.48 (1.17-1.88)	2.43 (1.92-3.08)	<0.001
	model 2		1.25 (0.98-1.58)	1.46 (1.15-1.85)	2.34 (1.84-2.97)	<0.001
Waist		1	1.22 (1.03-1.44)	1.82 (1.54-2.16)	2.74 (2.31-3.26)	<0.001
	model 1		1.01 (0.85-1.20)	1.17 (0.97-1.40)	1.45 (1.19-1.77)	<0.001
	model 2		1.01 (0.85-1.20)	1.16 (0.97-1.40)	1.44 (1.18-1.75)	0.0009
TG		1	1.53 (1.23-1.89)	2.13 (1.74-2.62)	3.96 (3.24-4.83)	<0.001
	model 1		1.38 (1.11-1.72)	1.69 (1.36-2.11)	2.87 (2.93-3.59)	<0.001
	model 2		1.38 (1.12-1.73)	1.69 (1.36-2.11)	2.86 (2.28-3.58)	<0.001
HDL		1	1.15 (0.96-1.38)	1.17 (0.98-1.40)	1.62 (1.36-1.93)	<0.001
	model 1		1.15 (0.96-1.37)	1.16 (0.97-1.39)	1.60 (1.34-1.91)	<0.001
	model 2		1.15 (0.95-1.37)	1.15 (0.95-1.39)	1.55 (1.27-1.90)	<0.001
BP		1	1.53 (1.26-1.84)	2.83 (2.36-3.40)	3.83 (3.20-4.60)	<0.001
	model 1		1.01 (0.82-1.24)	1.13 (0.92-1.40)	1.08 (0.87-1.34)	0.5824
	model 2		1.01 (0.82-1.24)	1.14 (0.92-1.40)	1.08 (0.87-1.34)	0.5759
GLU		1	2.04 (1.46-2.87)	2.73 (1.97-3.78)	5.82 (4.28-7.91)	<0.001
	model 1		1.61 (1.14-2.27)	1.60 (1.13-2.27)	2.89 (2.06-4.06)	<0.001
	model 2		1.60 (1.14-2.27)	1.58 (1.12-2.24)	2.80 (1.99-3.93)	<0.001

a: model 1, Odds ratio adjusted for age, nationality, smoking use, and alcohol consumption.

b: model 2, Odds ratio adjusted for model 1 plus hs-CRP.

## Discussion

In this analysis, we confirmed that there was a positive association between higher ferritin levels and the prevalence of MetS or MetS components in different sex groups. This association was independent of age, nationality, location, smoking status, and alcohol consumption. To our knowledge, this is the biggest population-based study to describe ferritin concentration distribution in Chinese population. Our results suggested that the body iron overload is an important independent risk factor for relevant diseases of MetS in Chinese population.

One recent study by Park et al [40] reported that elevated serum ferritin levels were independently associated with future development of MetS during the 5-year follow-up period. Several previous cross-sectional studies have reported the association between iron stores and individual components of metabolic syndrome, including hypertension [16], dyslipidemia [17,18], elevated fasting insulin and blood glucose [38], and central adiposity [23] in western countries. Few data are available in China yet. Studies have shown that serum ferritin concentrations differ significantly according to age, sex, geographic location, and race [27,28]. Harris and colleagues [39] found Asian men and women have higher adjusted mean serum ferritin concentrations compared with their white counterparts. Liang Sun [11] reported a strong positive association between elevated plasma ferritin concentrations and the risks of type 2 diabetes, impaired fasting glucose, and MetS among participants recruited from Beijing and Shanghai only. Studies in other areas of China had inconsistent or conflicting findings among Chinese men [25,26].

We found a positive association between ferritin levels and the prevalence of MetS in both men and women group. Ferritin levels also correlated with individual components of MetS, particularly serum TG and plasma glucose, as well as markers of insulin resistance. One of the advantages of our study is that our data have much larger sample size and can be a better representative sample of China. The participants in this study were recruited from nine provinces, including urban, suburban, towns and village population, representing a wider variety of Chinese. We have a few limitations as well. This cross-sectional analysis does not examine temporal changes in MetS components owing to the fact that biomarker data were only collected in the 2009 round of the CHNS. And we are not able to distinguish type 1 from type 2 diabetes in our survey.

In our study, we defined MetS based on the updated NCEP-ATPIII for Asian-Americans. Although some of the MetS components might be influenced by environment and diet, we have not found the authoritative criteria for Asian population to use.

In summary, moderately elevated iron levels were associated with an increased prevalence of the metabolic syndrome and markers of insulin resistance. These associations were evident at moderately elevated iron levels. Given the high prevalence of elevated iron stores, especially in older ages, prospective studies are needed to determine whether moderately elevated iron stores precede the development of insulin resistance and contribute to the increased risk associated with it.
